# When Pills Get a Pass and Lifestyle Treatments Don't: Misapplication of Phase III Logic to Phase IV Evaluation in Health Care

**DOI:** 10.1111/jep.70376

**Published:** 2026-02-10

**Authors:** Benno Krachler, Margareta Norberg, Lars Weinehall, Urban Janlert, Margareta Kristenson

**Affiliations:** ^1^ Department of Epidemiology and Global Health Umeå University Umeå Sweden; ^2^ Region Västernorrland Livsstilsmedicin Österåsen Sollefteå Sweden; ^3^ Department of Public Health and Clinical Medicine Umeå University Umeå Sweden; ^4^ Department of Health, Medicine and Caring Sciences, Division of Society and Health Linköping University Sweden

A recent BMJ article raised concerns about the growing burden of preventive responsibilities placed on general practitioners (GPs) [1]. One of the authors is based in Sweden, where the article sparked debate over the country's model of health‐promoting consultations known as *riktade hälsosamtal* (literally: ‘targeted health conversations’). These consultations include a structured, personalized dialogue grounded in *Motivational Interviewing* and designed to support health‐promoting behaviours. The dialogue is based on self‐reported health behaviours (tobacco use, alcohol, dietary habits, and physical activity) and measurements of physiological risk markers for cardiovascular disease (blood glucose, lipids, body weight, waist circumference, and blood pressure) presented in a pedagogical diagram. These consultations, offered to all citizens at ages 40, 50, and 60, are conducted at the nearest primary care centre and form part of broader public health strategies enhancing health behaviour change [2].

Representatives of SFAM, the Swedish Association of General Practitioners, questioning the appropriateness of *riktade hälsosamtal* within the context of an already stretched primary care system, joined the discussion in professional media [3, 4]. A recurring theme was the demand for evidence from randomised controlled trials (RCTs) and the call for applying similar standards of evidence to both pharmaceuticals such as statins and to *riktade hälsosamtal* [4].

## 1 The Illusion of Certainty: What RCTs of Drugs Really Show

This view reflects a common assumption: that prescribing statins is firmly grounded in robust evidence from RCTs. But what such evidence actually demonstrates is that, under controlled conditions with carefully selected patients, the active agent—a statin—reduces cardiovascular risk. It does not show that prescribing statins in routine primary care consistently leads to the same outcome. Each step between consultation and clinical effect—correct indication, appropriate prescribing, patient understanding, medication dispensing, initiation, adherence, and long‐term persistence—is subject to real‐world variation.

This variation arises from a range of individual and contextual factors—including, but not limited to, socioeconomic status, health literacy, and competing life demands on the part of the patient; primary care doctors’ clinical judgement, willingness, and ability to communicate and engage; and, at the system level, contextual factors such as fragmented care structures and limited consultation time. Adding to this is the clinical complexity posed by comorbidity and multimorbidity, which challenge the straightforward application of single‐condition guidelines and affect both clinical decisions and patient capacity to follow through.

Further compounding these challenges are issues of limited availability of prescription drugs—such as supply shortages or forced substitutions due to stockouts or brand discontinuities—which further disrupt continuity of care and undermine treatment fidelity. Its effects—missed prescriptions, suboptimal dosing, poor adherence, and early discontinuation—accumulate along the implementation chain.

The traditional drug development pathway is outlined in Table 1.

**Table 1:** The traditional drug development pathway. This table outlines the typical phases of drug development, from preclinical testing through post‐marketing surveillance. Each phase is defined by its primary purpose, the population involved, and the typical study setting. While trials in Phases I–III are focused on efficacy and safety under controlled conditions, Phase IV evaluates real‐world effectiveness after regulatory approval.

Overview of clinical trial phases				
Phase	Primary focus	Key question	Typical setting	Population
Phase I	Safety and dosage	Is it safe? What's the right dose?	Tightly controlled, research unit	Small group of healthy volunteers or patients
Phase II	Preliminary efficacy + safety	Does it show signs of working? Are there short‐term side effects?	Controlled, selective clinical setting	100‐300 selected patients
Phase III	Confirmatory efficacy	How well does it work under ideal conditions?	Controlled, often multicenter	Large sample of patients from selected sites
Phase IV	Real‐world effectiveness/surveillance	How does it perform in the real world? Ar there rare/long‐term side effects?	Routine clinical use	Broad, real‐world population

*Note:* While Phases I–III operate under controlled trial conditions to test efficacy and safety, Phase IV is the only phase where real‐world effectiveness and implementation challenges are assessed.

Phase I to III clinical trials, which precede regulatory approval, are expressly designed to minimize real‐world variation: they employ specific recruitment sites, strict inclusion criteria, intensive monitoring, and comprehensive support for both patients and providers [5]. Yet once a drug is registered—such as by the FDA or EMA—this orchestrated framework falls away. The intervention exits the confines of selected trial sites, and, as it enters the variability of routine care, an implementation gap opens. This gap reflects unrealized health benefits due to real‐world variation in delivery, uptake, and context. While literature on other effects of real‐world variation is scarce, adherence rates of just 50% suggest that more benefit is lost in translation than is realised in practice [6].

## 2 It's Not the Behaviour That Needs Testing, but the Delivery

**Figure 1:**
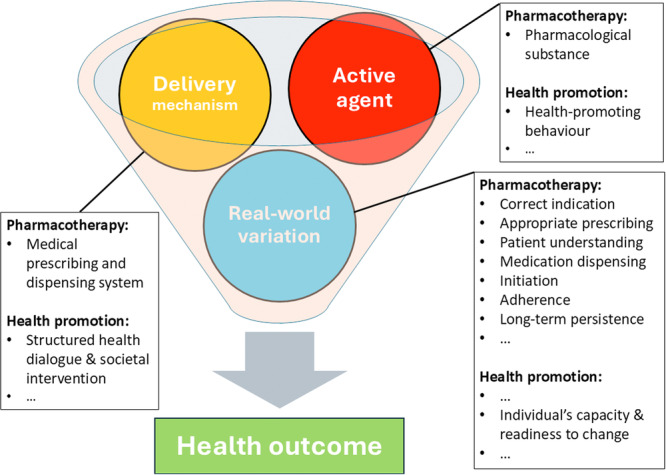
Intervention core‐components. The Delivery mechanism modulates access to and engagement with the Active agent, thereby influencing to what degree the intended Health outcome is achieved given a specific configuration of Real‐world variation. While trial protocols aim to optimize conditions for demonstrating the active agent's effect by minimizing real‐world variation, it is ultimately the configuration of real‐world variation that shapes health outcomes in routine care.

Interventions targeting health‐related behaviours involve the same core components as pharmacological interventions: an active agent (the desired behaviours), a delivery mechanism (structured support), and exposure to sources of real‐world variation (Figure 1).

The relevance of sustained health‐promoting behaviours for long‐term health outcomes is well established: of the ten leading causes of disease burden and premature death, nine are either health‐related behaviours (diet, alcohol use, drug use, smoking, physical inactivity) or behaviour‐related risk factors (high blood pressure, elevated BMI, raised fasting glucose, and high total cholesterol) [7].

Therefore, unlike pharmaceuticals, the beneficial effects of behavioural ‘active agents’—such as a tobacco‐free lifestyle or regular physical activity—do not require testing in isolation under tightly controlled conditions, nor long‐term surveillance for unforeseen side effects. However, the principal sources of real‐world variation are broadly similar. They include many of the same contextual and systemic factors that affect pharmacological therapies, such as socioeconomic conditions, trust in healthcare providers, and broader societal context. But behavioural interventions are additionally—and often more profoundly—shaped by individual‐level dynamics that influence a person's willingness, readiness, and capacity to change. These are not fixed traits, but the outcome of a complex interplay of psychological and contextual influences—including lived experience, support systems, and opportunity structures such as access to healthy food or workplace flexibility.

These inherently heterogeneous conditions necessitate a context‐sensitive adaptation of strategies. Real‐world variation, rather than noise to be cancelled out, is the primary challenge to be addressed [8]. RCTs—designed to isolate the effect of an active agent under tightly controlled conditions—are ill‐equipped to evaluate implementation strategies whose success depends on engaging with, not retreating from, such complexity. For behavioural interventions, what requires study is not the efficacy of the desired behaviour, but which delivery mechanisms work for whom, and under what real‐world conditions.

## 3 Efficacy, Effectiveness, and the Implementation Gap

This brings us to the heart of the issue: for any intervention, we must distinguish between two dimensions of outcome:

- Efficacy describes how well an intervention works under ideal, controlled conditions — for example, in randomized trials with carefully selected participants and implementation resources that are not typically available in routine care.
- Effectiveness refers to what the intervention actually achieves in real life — when used in routine care or community settings, where delivery, uptake and contexts vary.

**Figure 2:**
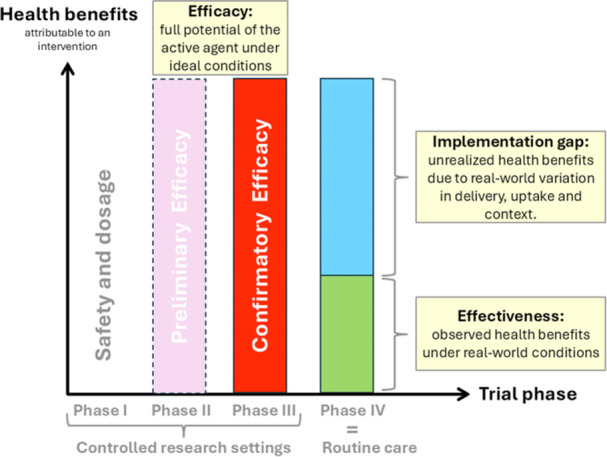
Clinical trial phases and the implementation gap. Efficacy represents the health benefit achievable by the active agent under ideal and controlled conditions (phase III). Effectiveness, as assessed in phase IV, reflects what is achieved in real‐world practice. The gap between the two — the implementation gap — captures the portion of potential benefit lost (unachieved) due to real‐world variation in delivery, uptake, and contextual factors. This illustrates how real‐world conditions shape outcomes, even when efficacy is high.

The difference between these two is known as the implementation gap — the potential health benefits that are lost/remain unachieved when an intervention moves from controlled research settings to routine care (Figure 2). Without this distinction, we risk applying asymmetric standards: expecting one type of interventions to prove their value in routine care (effectiveness), while accepting others based on results from trials in controlled research settings (efficacy).

## 4 The Double Standard in Evaluating Behavioural Interventions

Randomized controlled trials are often discussed as a single category, yet it is essential to distinguish between explanatory (phase III) RCTs, designed to test the efficacy of an active agent under controlled conditions, and pragmatic (phase IV) RCTs, which evaluate interventions as delivered in routine care. For complex behavioural interventions, pragmatic RCTs necessarily test a compound of active agent, delivery mechanism, and context. Internal contextual variability increases the risk of false‐negative results, while external contextual variation makes positive findings difficult to generalise. Thus, when critics argue that *riktade hälsosamtal* lack scientific support because no RCT has shown improved health outcomes, they are implicitly requesting phase III‐level answers for phase IV‐level questions. That is, they expect real‐world health outcomes to be demonstrated under controlled research conditions—even though effectiveness is contingent on adaptive, context‐sensitive implementation across diverse patients, settings, and societal contexts.

This standard is rarely applied to pharmacological interventions. For example, we routinely accept phase III data on statins as sufficient proof of benefit, even though real‐world adherence, prescribing patterns, and patient engagement—i.e. the very domain of phase IV—introduce a substantial implementation gap. Judged by the same effectiveness criteria, one might argue that statins often fall short in practice [9, 10], while *riktade hälsosamtal*, evaluated at the population level, do deliver, as demonstrated by evidence of both mortality reduction and cost‐effectiveness [11, 12, 13].

## 5 Rethinking Evidence Standards: From Phase III Logic to Phase IV Reality

This failure to distinguish between phase III efficacy and phase IV effectiveness is not merely technical—it reflects a deeper conceptual blind spot that leads to asymmetric standards: while drugs are prescribed on the basis of efficacy data, behavioural interventions are expected to prove effectiveness in RCTs—trials that, by design, depend on cancelling out real‐world variation to isolate causal effects. Even when this demanding and resource‐intensive task is undertaken, such an RCT must be carefully engineered to minimise implementation variability in order to detect an effect. The trade‐off is reduced external validity; the findings then tell us little about how the intervention would perform in routine practice. As a result, we risk discarding strategies that work—not because they are ineffective, but because they fail to conform to a misapplied evidentiary ideal.

If we intend to apply evidentiary standards based on the traditional drug development pathway to evaluate different types of interventions, we must begin by distinguishing between two elements that are often conflated: the active agent—such as a drug or a behaviour—and the delivery mechanism through which it is introduced, supported, and maintained in real‐world settings. Only once this distinction is made can we clarify what exactly we aim to evaluate—whether it is the efficacy of the active agent under controlled research conditions (as in phase III), or the effectiveness of a delivery mechanism in routine care (as in phase IV). Evidentiary standards must be set accordingly, so that the robustness of the available evidence can be fairly judged—regardless of whether the intervention takes the form of a pharmaceutical prescription, a surgical procedure, or a conversation about health in primary care.

## 6 Alternative Phase IV Evaluation Approaches

While the main argument of this paper is conceptual—highlighting the need to align evaluation methods with the developmental phase of an intervention—it is worth briefly noting that several frameworks have emerged for assessing real‐world effectiveness in complex public health settings. Dynamic cohort approaches are often the most feasible and informative for assessing population‐based preventive interventions in real‐world settings. Such designs allow evaluation of outcomes as programmes evolve and expand, while maintaining naturalistic variation in exposure and context. The recently published *systematic review of the Swedish model of health dialogues* [14] exemplifies this approach: six of the seven included studies employed observational, dynamic cohort designs with long follow‐up, demonstrating significant reductions in all‐cause and cardiovascular mortality as well as improvements in major risk factors. Other approaches such as realistic evaluation [15], hybrid effectiveness–implementation designs [16], or stepped‐wedge cluster randomized trials [17] represent pragmatic alternatives that integrate methodological rigour with contextual relevance. Each offers tools for exploring how and why interventions succeed or fail under routine conditions, focusing on mechanisms, context, and implementation processes rather than isolated efficacy. Although these methods fall outside the central scope of this paper, they exemplify the shift from phase III logic toward phase IV reality—where the goal is not to eliminate real‐world variation, but to understand and work productively within it.

## Author Contributions

Benno Krachler and Margareta Kristenson conceived the idea for the article, and Benno Krachler wrote the initial draft. Margareta Norberg, Lars Weinehall, Urban Janlert, and Margareta Kristenson contributed with clinical, policy and methodological input, and helped revise the manuscript.

## Funding

The authors received no specific funding for this work.

## Conflicts of Interest

The authors declare no conflicts of interest.

## Data Availability

Data sharing not applicable to this article as no datasets were generated or analysed during the current study.

## References

[1] S. A. Martin , M. Johansson , I. Heath , R. Lehman , and C. Korownyk , “Sacrificing Patient Care for Prevention: Distortion of the Role of General Practice,” BMJ 388 (2025): e080811, 10.1136/bmj-2024-080811. 39837625

[2] M. Norberg , S. Wall , K. Boman , et al., “The Vasterbotten Intervention Programme: Background, Design and Implications,” Global Health Action 3 (2010): Article: 4643, 10.3402/gha.v3i0.4643. PMC2844807 20339479

[3] M. Skogström , M. Wibom , M. Neumann , et al. RCT är inte svaret på allt – låt inte det bästa bli det godas fiende [RCTs Are Not the Answer to Everything – Don't let the Best Become the Enemy of the Good]. 2024. accessed, June 21, 2025, https://lakartidningen.se/opinion/debatt/2024/11/rct-ar-inte-svaret-pa-allt-lat-inte-det-basta-bli-det-godas-fiende/.

[4] A. Niklasson , O. Lindfors , D. Gyll , et al., “Primärvården Bör Återgå Till Sitt Kärnuppdrag – Att Vårda Sjuka [Primary Care Should Return to Its Core Mission – Caring for the Sick],” Läkartidningen 122 (2025): 25019. 40200891

[5] R. C. Armitage , “Pre‐Screening in Clinical Trials: Incentives, Behaviours, Consequences,” Journal of Evaluation in Clinical Practice 31, no. 5 (2025): e70231, 10.1111/jep.70231. 40741860 PMC12312072

[6] World Health Organization. Adherence to Long‐Term Therapies. Evidence for Action. In: Geneva WHO, ed., 2003.

[7] T. Vos , S. S. Lim , C. Abbafati , et al., Collaborators. GBDDI ., “Global Burden of 369 Diseases and Injuries in 204 Countries and Territories, 1990‐2019: a Systematic Analysis for the Global Burden of Disease Study 2019,” Lancet 396, no. 10258 (2020): 1204–1222, [published Online First: 2020/10/19] 10.1016/S0140-6736(20)30925-9. 33069326 PMC7567026

[8] J. P. Sturmberg and M. Mercuri , “Every Problem Is Embedded in a Greater Whole,” Journal of Evaluation in Clinical Practice 31, no. 1 (2025): e14139. [published Online First: 2024/09/23] 10.1111/jep.14139. 39308191

[9] C. Brotons , “The Challenge of Therapy Adherence in Clinical Practice,” European Journal of Preventive Cardiology 30, no. 2 (2023): 147–148, [published Online First: 2022/09/06] 10.1093/eurjpc/zwac197. 36062950

[10] R. Elhiny , L. M. O'keeffe , E. O. Bodunde , S. Byrne , M. Donovan , and M. Bermingham , “Goal Attainment, Medication Adherence and Guideline Adherence in the Treatment of Hypertension and Dyslipidemia in Irish Populations: A Systematic Review and Meta‐Analysis,” International Journal of Cardiology. Cardiovascular Risk and Prevention 24 (2025): 200364. [published Online First: 2025/01/04] 10.1016/j.ijcrp.2025.200364. 39877073 PMC11773485

[11] Y. Blomstedt , M. Norberg , H. Stenlund , et al., “Impact of a Combined Community and Primary Care Prevention Strategy on All‐Cause and Cardiovascular Mortality: A Cohort Analysis Based on 1 Million Person‐Years of Follow‐Up in Västerbotten County, Sweden, During 1990–2006,” BMJ Open 5, no. 12 (2015): e009651, 10.1136/bmjopen-2015-009651. PMC4691769 26685034

[12] L. Lindholm , A. Stenling , M. Norberg , H. Stenlund , and L. Weinehall , “A Cost‐Effectiveness Analysis of a Community Based CVD Program in Sweden Based on a Retrospective Register Cohort,” BMC Public Health 18, no. 1 (2018): 452, [published Online First: 20180404] 10.1186/s12889-018-5339-3. 29618323 PMC5885416

[13] H. Lingfors and L. G. Persson , “All‐Cause Mortality Among Young Men 24‐26 Years After a Lifestyle Health Dialogue in a Swedish Primary Care Setting: A Longitudinal Follow‐Up Register Study,” BMJ Open 9, no. 1 (2019): e022474. [published Online First: 2019/01/29] 10.1136/bmjopen-2018-022474. PMC6352829 30696668

[14] M. Borjesson , M. Kristenson , L. Jerdén , and Y. Forsell , “The Swedish Model of Health Dialogues, a Combined Individual‐ and Community‐Based Primary Preventive Program for Cardiovascular Disease, Is Associated With Reduced Mortality: A Systematic Review,” BMC Public Health 25 (2025): 3288, 10.1186/s12889-025-24353-0. 41039382 PMC12490140

[15] P. R. T. Nicholas , Realistic Evaluation (SAGE Publications Ltd, 1997).

[16] G. M. Curran , M. Bauer , B. Mittman , J. M. Pyne , and C. Stetler , “Effectiveness‐Implementation Hybrid Designs: Combining Elements of Clinical Effectiveness and Implementation Research to Enhance Public Health Impact,” Medical Care 50, no. 3 (2012): 217–226, 10.1097/MLR.0b013e3182408812. 22310560 PMC3731143

[17] F. Li and R. Wang , “Stepped Wedge Cluster Randomized Trials: A Methodological Overview,” World Neurosurgery 161 (2022): 323–330, 10.1016/j.wneu.2021.10.136. 35505551 PMC9074087

